# Supplementary material for: Characterizing Molecule–Metal Surface Chemistry with Ab Initio Simulation of X-ray Absorption and Photoemission Spectra
Source: J Phys Chem C Nanomater Interfaces. 2023 Jan 23;127(4):1870–80. doi: 10.1021/acs.jpcc.2c06996 (PMC9900587; doi:10.1021/acs.jpcc.2c06996)
Supplement: Supplementary file 1 — jp2c06996_si_001.pdf [file jp2c06996_si_001.pdf]

Supplementary Information: Characterizing Molecule-Metal Surface Chemistry with Ab-Initio Simulation of X-ray Absorption and Photoemission Spectra

Samuel J. Hall,[†,‡] Benedikt P. Klein,[†,¶] and Reinhard J. Maurer[*,†]

†*Department of Chemistry, University of Warwick, Gibbet Hill Road, Coventry, CV4 7AL, United Kingdom*

‡*MAS Centre of Doctoral Training, Senate House, University of Warwick, Gibbet Hill Road, Coventry, CV4 7AL, United Kingdom*

¶*Diamond Light Source, Harwell Science and Innovation Campus, Didcot, OX11 0DE, United Kingdom*

E-mail: r.maurer@warwick.ac.uk

Supplementary Information

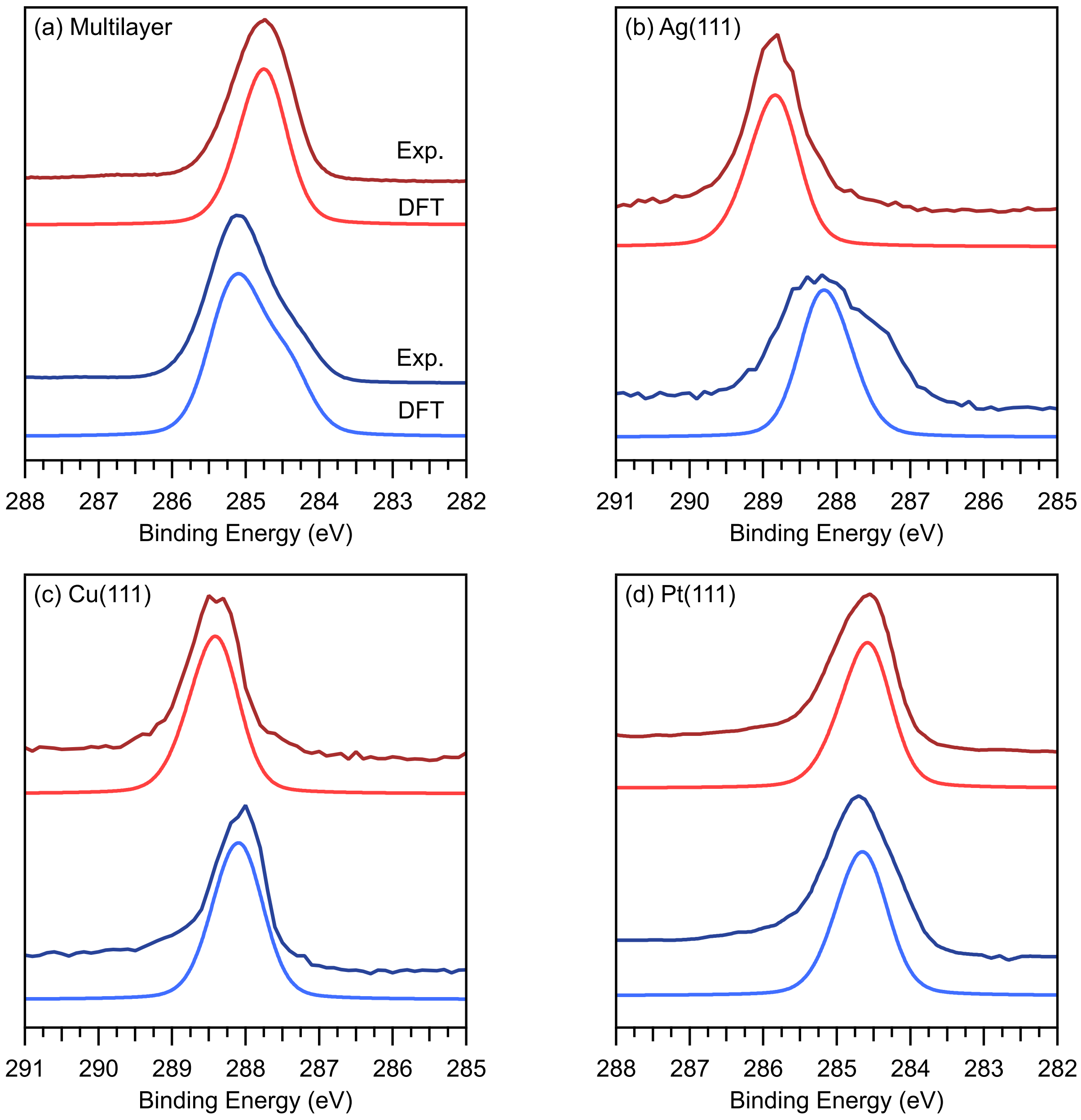

Figure S1: Comparison of computational results to experimentally recorded C 1s XPS spectra of naphthalene (in red) and azulene (in blue) in a multilayer sample (a), adsorbed on an Ag(111) surface (b), on Cu(111) in (c), and on Pt(111) in (d). Experimental data previously published in Refs. [1–3]. Experimental spectra shown on top in darker shade whilst below in lighter shade are the DFT simulated results. A shift was applied to the computational results to match the experimental energy scale.

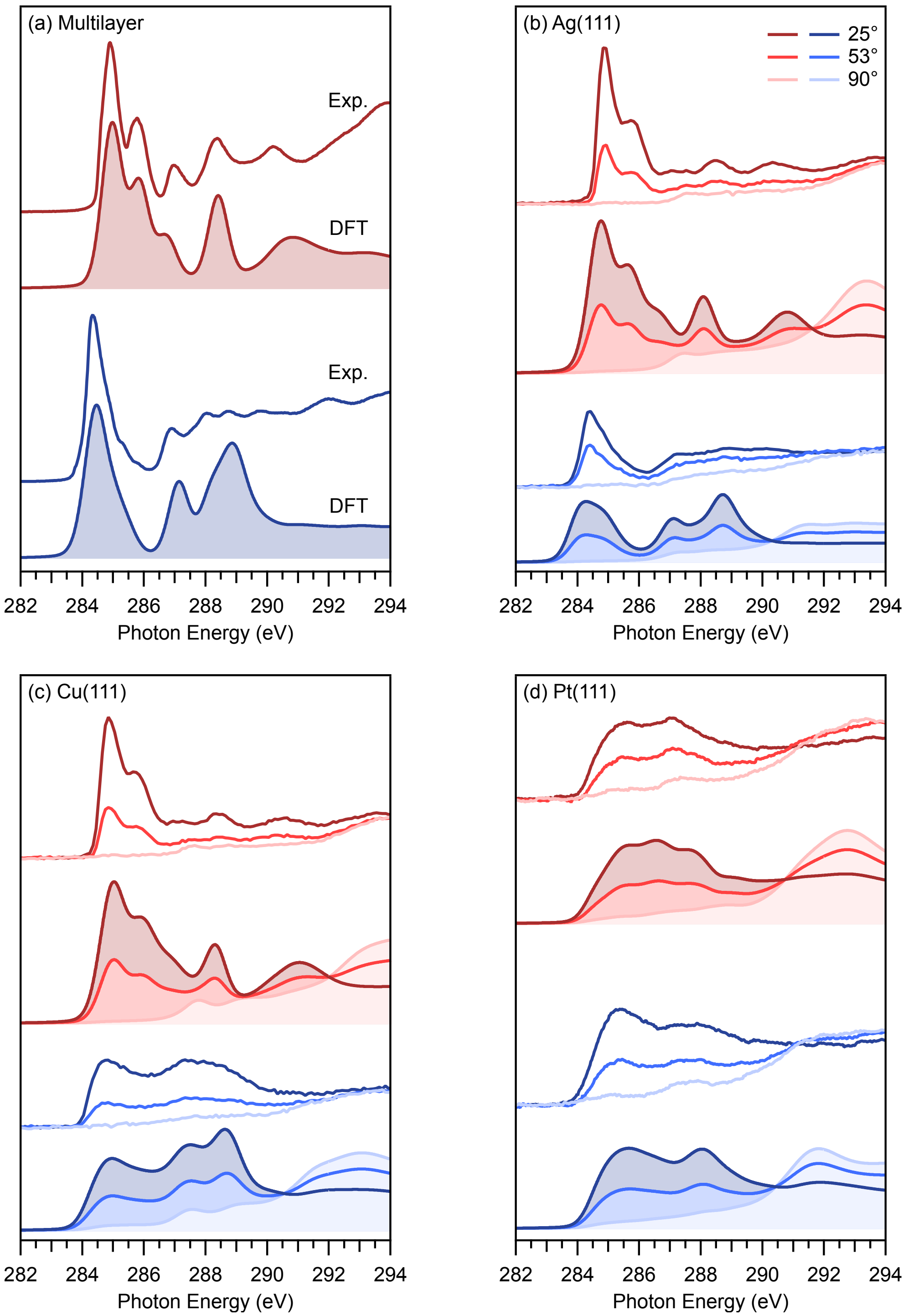

Figure S2: Comparison of computational results to experimentally recorded C K-edge NEXAFS spectra of naphthalene (red) and azulene (blue) previously published in Refs. [1–3]. (a) shows the spectra of a multilayer sample recorded at a 25° incidence angle. (b) are spectra for the molecule adsorbed on an Ag(111) surface, (b) adsorbed on Cu(111), and (d) adsorbed on Pt(111). Spectra for three different incidence angles of 25°, 53°, and 90° are shown from darker to lighter line shades. Experimental spectra are shown at the top with simulated DFT spectra below as shaded spectra. A shift was applied to the computational results to match the experimental energy scale.

Table S1: Calculated net charge transfer of all metal adsorbed systems investigated using various charge analysis methods and electronic structure codes . All values are given in e, a negative value means electrons are transferred from the surface to the molecule. Bader and DOS charges for all systems, as well as Hirshfeld values for Pt systems, have previously been reported in Refs. [2, 3].

Method	Nt/Ag	Az/Ag	Nt/Cu	Az/Cu	Nt/Pt	Az/Pt
DOS (CASTEP)	-0.05	-0.21	-0.13	-1.39	-1.70	-1.60
Hirshfeld (CASTEP)	-0.07	-0.11	-0.05	-0.35	-0.32	-0.31
Hirshfeld (VASP)	-0.04	-0.07	-0.01	-0.25	-0.20	-0.21
It-Hirshfeld (VASP)	-0.14	-0.19	-0.12	-0.60	-0.86	-0.84
Bader (ADF-BAND)	+0.06	+0.01	+0.06	-0.49	+0.03	+0.02

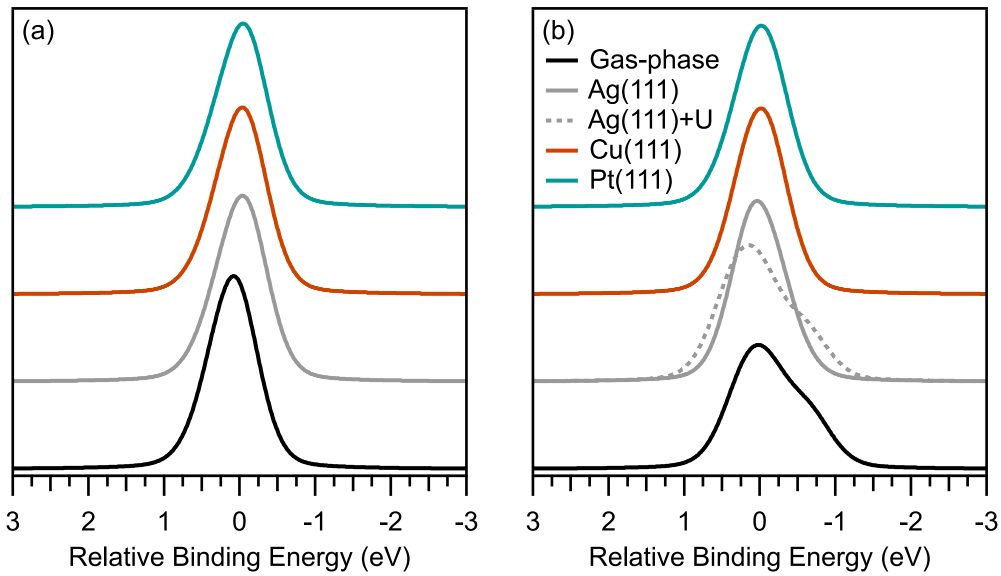

Figure S3: Comparison of XPS spectra for naphthalene (a) and azulene (b) in the gas-phase (black) previously published in Ref. [1], and adsorbed on three metal surfaces, Ag(111) (gray), Cu(111) (orange) and Pt(111) (green). Spectra of the metal adsorbed systems have been aligned to the center of mass.

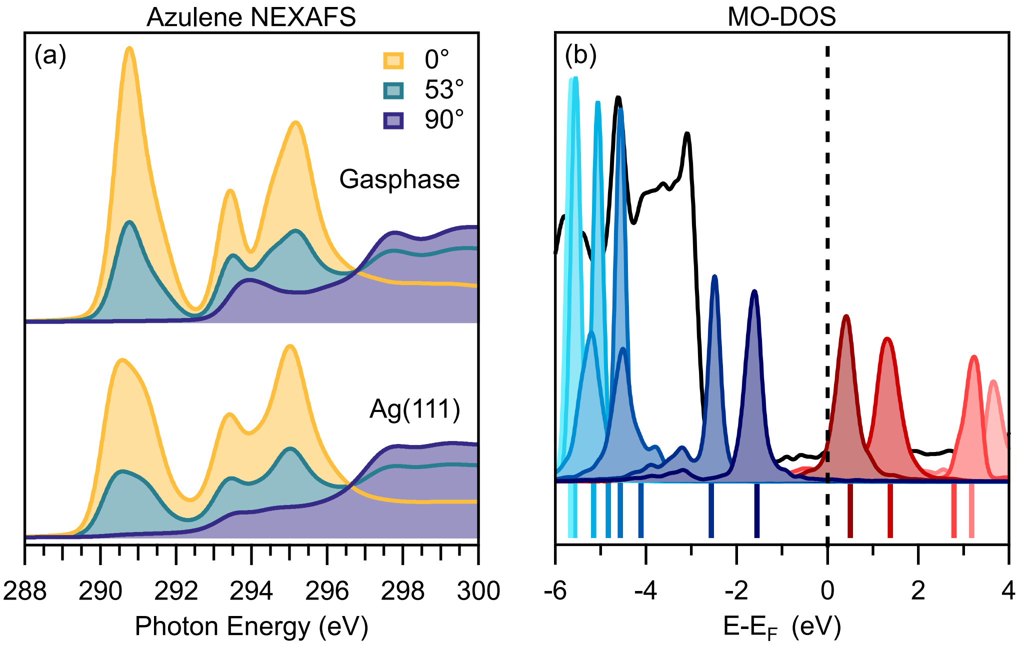

Figure S4: (a) DFT calculated NEXAFS spectra of azulene in two different systems. Gas-phase (top) and the physisorbed Az/Ag(111) (bottom). Three different incidence angles are shown of 0° (yellow), 53° (green) and 90° (blue). (b) shows DFT density of states (DOS) of the metal adsorbed system. Total DOS is shown in black and Fermi level shown with dashed line, contributions from orbitals scaled for ease of viewing gas-phase. Labeled in blue orbitals represent the HOMOs and red the LUMOs with the lighter shade moving lower or higher in energy respectively. Colored lines at the bottom of the graph represent gas-phase orbitals shifted by 3.3 eV to aligned with the metal adsorbed system. All data was previously published in Ref. [2], except the 0° NEXAFS spectra.

Table S2: Absolute energies and intensities of first 3 peaks seen in NEXAFS spectra of azulene gas-phase and on Ag(111) and Cu(111) surfaces for spectra simulated with a 0° incidence angle. All intensities have been normalized to the leading Az/Gas peak.

Peak	Az/Gas		Az/Ag(111)		Az/Cu(111)	
	Photon Energy (eV)	Intensity	Photon Energy (eV)	Intensity	Photon Energy (eV)	Intensity
1	290.77	1.00	290.58	0.65	290.97	0.43
2	293.43	0.48	293.42	0.45	293.51	0.50
3	295.17	0.73	295.02	0.70	294.62	0.59

Table S3: Relative intensities of the first three peaks in the NEXAFS spectrum of azulene in the gas-phase, Az/Ag(111), and Az/Cu(111), normalised to the intensity of the third peak in each spectrum.

Peak	Az/Gas	Az/Ag(111)	Az/Cu(111)
1	1.37	0.93	0.73
2	0.66	0.64	0.84
3	1.00	1.00	1.00

Table S4: Relative intensities of the first two peaks in the NEXAFS spectrum of Az/Ag(111) and Az/Cu(111), first normalised to the third peak in each spectrum then to the intensity of the corresponding peak intensity of gas-phase azulene (Table S3).

Peak	Az/Ag(111)	Az/Cu(111)
1	0.68	0.53
2	0.97	1.27
3	1.00	1.00

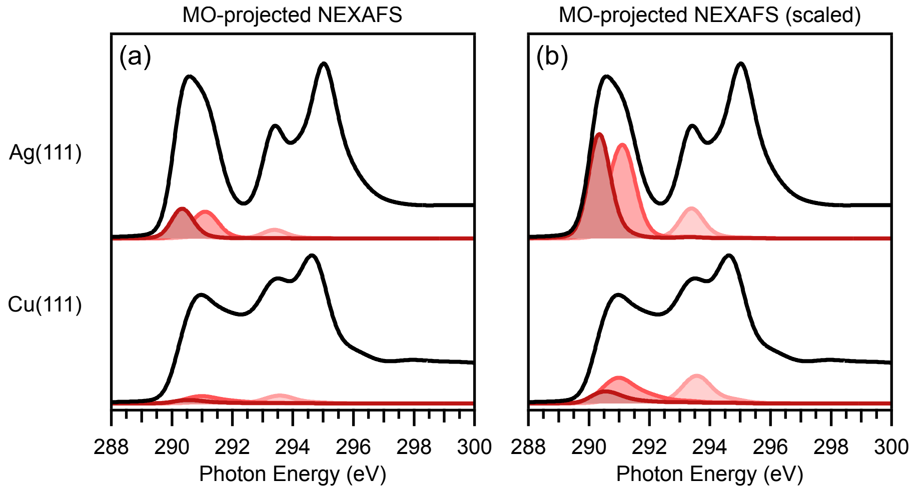

Figure S5: MO-projected NEXAFS of Az/Ag(111) (top) and Az/Cu(111) (bottom) using the simulated 0° spectra. (a) presents the MO-decomposition as calculated and (b) scales contributions for both Ag(111) and Cu(111) by the same amount for ease of viewing.

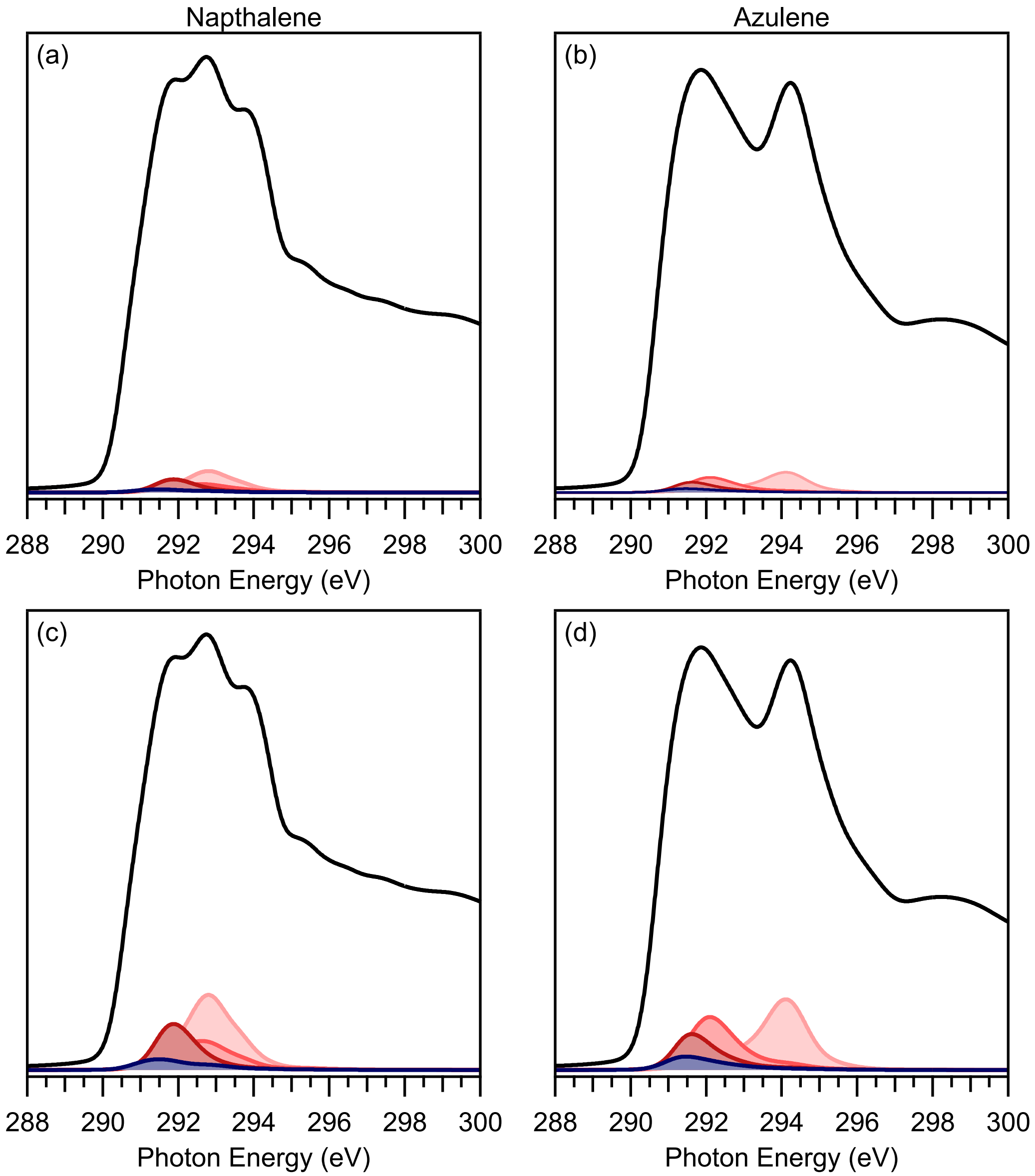

Figure S6: MO decomposition of 0° NEXAFS of the type III systems of naphthalene, (a), and azulene, (b), adsorbed on Pt(111). (c) and (d) show the same decomposition as above but with the MO orbitals scaled for ease of viewing. Previously published in Ref. [3], recalculated with updated methodology.

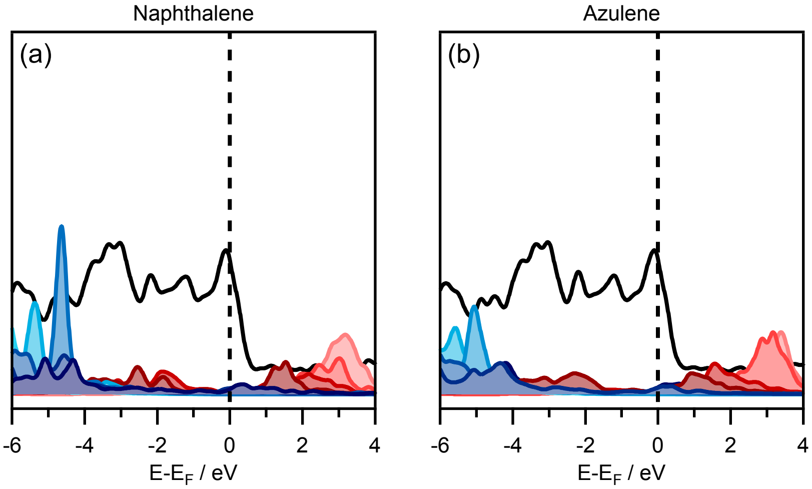

Figure S7: DFT density of states DOS with scaled MO projections for naphthalene (a) and azulene (b) adsorbed on a Pt(111) surface. The total DOS is shown in black with Fermi level shown as dashed vertical line. Contributions in blue represent projected occupied states, while contributions in red represent unoccupied states. Data previously published in Ref. [3].

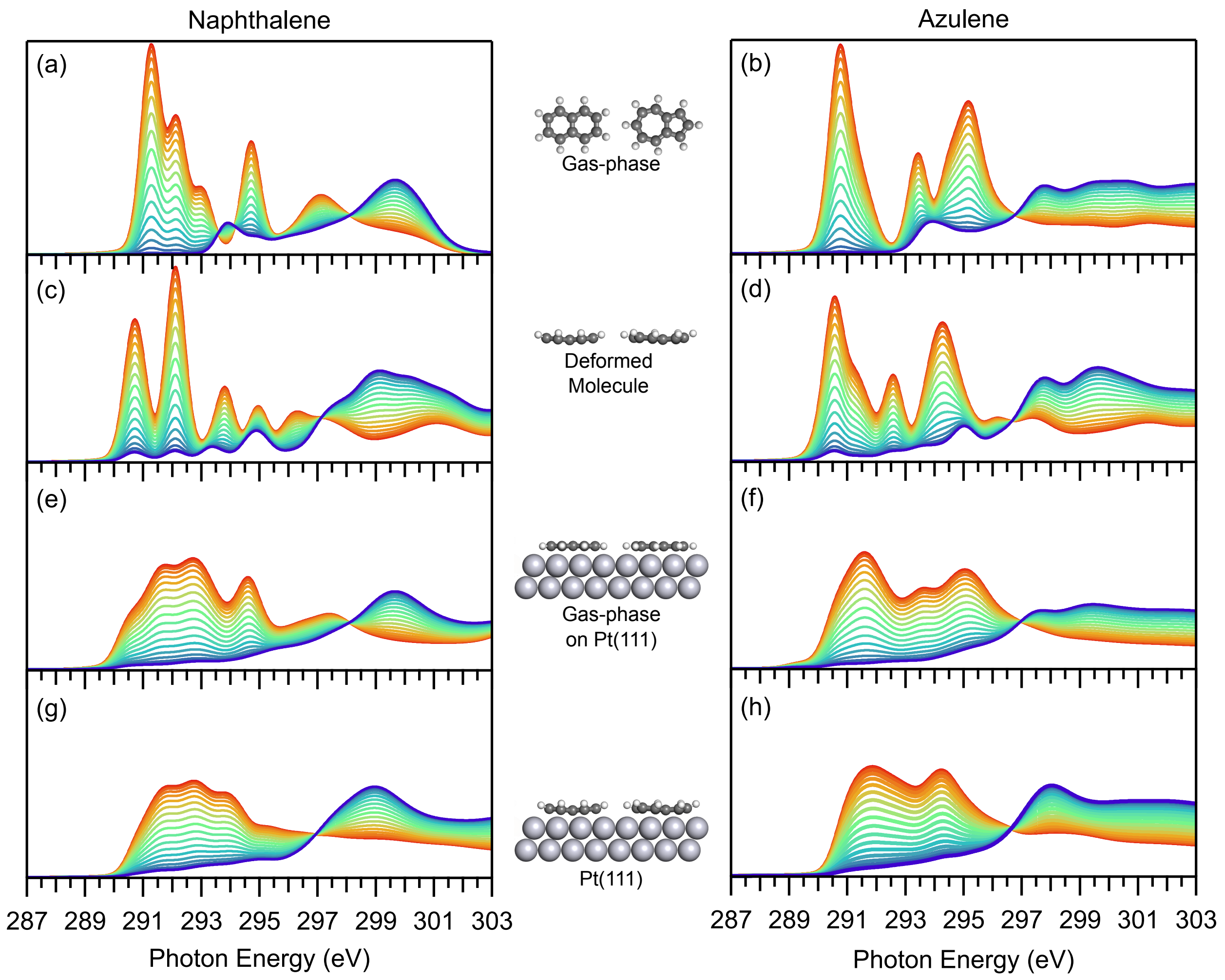

Figure S8: Simulated NEXAFS spectra of naphthalene (left) and azulene (right). Dichroism is depicted by showing spectra with varying incidence angles from normal incidence 90° (blue) to grazing incidence 0° (red). (a,b) show the spectra for the gas-phase molecule, (c,d) for the molecule in the gas phase, but with the adsorption induced deformation, (e,f) for the planar gas-phase molecule adsorbed on Pt with the proper adsorption site and height, and (g,h) for the fully relaxed structure of the molecule adsorbed on Pt. Next to all spectra is a model visualizing the corresponding structure.

References

(1) Klein, B. P.; van der Heijden, N. J.; Kachel, S. R.; Franke, M.; Krug, C. K.; Greulich, K. K.; Ruppenthal, L.; Müller, P.; Rosenow, P.; Parhizkar, S., et al. Molecular Topology and the Surface Chemical Bond: Alternant Versus Nonalternant Aromatic Systems as Functional Structural Elements. *Phys. Rev. X* **2019**, *9*, 11030.

(2) Klein, B. P.; Morbec, J. M.; Franke, M.; Greulich, K. K.; Sachs, M.; Parhizkar, S.; Bocquet, F. C.; Schmid, M.; Hall, S. J.; Maurer, R. J., et al. The Molecule-Metal Bond of Alternant versus Nonalternant Aromatic Systems on Coinage Metal Surfaces: Naphthalene versus Azulene on Ag(111) and Cu(111). *J. Phys. Chem. C* **2019**, *123*, 29219–29230.

(3) Klein, B. P.; Harman, S. E.; Ruppenthal, L.; Ruehl, G. M.; Hall, S. J.; Carey, S. J.; Herritsch, J.; Schmid, M.; Maurer, R. J.; Tonner, R., et al. Enhanced Bonding of Pentagon–Heptagon Defects in Graphene to Metal Surfaces: Insights From the Adsorption of Azulene and Naphthalene to Pt(111). *Chem. Mater.* **2020**, *32*, 1041–1053.
